# Serum Galectin-3 levels and all-cause and cardiovascular mortality in maintenance hemodialysis patients: a prospective cohort study

**DOI:** 10.1186/s12882-021-02636-z

**Published:** 2022-01-03

**Authors:** Shuxin Liu, Qijun Wu, Shuang Zhang, Zhihong Wang, Hong Liu, Lanbo Teng, Ping Xiao, Yan Lu, Xuena Wang, Cui Dong, Jia Xiao, Jiayu Zhang

**Affiliations:** 1 Department of Nephrology, Dalian Municipal Central Hospital, No.826, Xinan Road, Dalian, Liaoning 116033 People’s Republic of China; 2 Department of Clinical Epidemiology, Shengjing Hospital of China Medical University, Shenyang, China

**Keywords:** Galectin-3, Mortality, Hemodialysis

## Abstract

**Background:**

Higher serum galectin-3 levels are related to adverse outcomes in different disease states. However, the association of galectin-3 with mortality in the maintenance hemodialysis (HD) population has not been fully described. Thus, we aimed to assess the predictive significance of galectin-3 for all-cause and cardiovascular (CV) mortality through a Chinese maintenance HD population.

**Methods:**

A prospective cohort study was conducted in five hundred and six patients with end-stage renal disease who underwent hemodialysis at Dalian Central Hospital before December 31, 2014. Serum galectin-3 levels were measured at baseline and classified as high (> 8.65 ng/ml) or low (≤ 8.65 ng/ml) according to the “X-tile” program. Primary and secondary outcomes were all-cause and CV mortality, respectively. Hazard ratios (HRs) and 95% confidence intervals (CIs) were calculated by the Cox proportional hazards regression models.

**Results:**

During the median follow-up of 60 months, there were 188 all-cause deaths and 125 CV deaths. Compared with maintenance HD population with galectin-3 ≤ 8.65 ng/ml, the adjusted HR for all-cause mortality among those with galectin-3 >  8.65 ng/ml was 1.59 (CI: 0.96–2.65, *p* = 0.07). Furthermore, multivariable analysis showed that maintenance HD patients with galectin-3 >  8.65 ng/ml had a 2.13-fold higher risk of CV death than those with galectin-3 ≤ 8.65 ng/ml (HR = 2.13, 95% CI 1.07–4.26).

**Conclusion:**

Galectin-3 is an independent predictor of CV mortality in maintenance HD patients.

## Introduction

End-stage renal disease (ESRD) constitutes a main clinical and public health problem due to its gradually increasing morbidity, high mortality and health care expenditures [1]. Hemodialysis (HD) is the most frequent modality of renal replacement therapy and a life-sustaining treatment for patients with ESRD, and its usage has continued to rise over the past 20 years [2, 3]. However, the majority of patients undergoing HD suffer from adverse physical symptoms, which are related to poor health-related quality of life [4–6]. Despite advances in HD technologies and medical care, maintenance HD patients remain at noticeably elevated risk of death, most frequently owing to cardiovascular (CV) disease [2]. For example, CV mortality in patients who are on dialysis is 10 to 20 times higher than in the general population [3]. The traditional contributing factors do not fully explain high mortality in the maintenance HD population, [7] therefore, novel and effective biomarkers to predict and improve survival in this population are urgently needed.

Galectin-3 is a soluble β-galactoside-binding lectin composed of 250 amino acids that has regulatory roles in fibrogenesis, inflammation, tissue repair, and cell proliferation [8–10]. Prior studies have suggested that higher galectin-3 concentrations may be related to the development of fibrosis of solid organ tissues, including heart, kidney, and liver [8, 9, 11]. Galectin-3 has been shown to play a role in the pathophysiology of heart failure (HF) by promoting myocardial fibrosis and inflammation and has become a significant predictor of HF [11–13]. Although galectin-3 assay has been used to assess prognosis of chronic HF and the prognostic utility of galectin-3 has been further demonstrated in other populations, [1, 14] its prognostic value in maintenance HD patients remains limited and inconsistent [1, 15–17]. Thus we aimed to investigate the prospective association between serum concentrations of galectin-3 and all-cause and CV mortality in maintenance HD patients.

## Material and methods

### Study population

We assessed the eligibility of all stable patients with ESRD who received HD treatment at Dalian Central Hospital before December 31, 2014. Inclusion criteria were 18 years or older and at least three months of HD treatment. Patients aged 80 years or older or with acute kidney injury or active malignancy were excluded. Finally, 506 maintenance HD patients who met the inclusion and exclusion criteria were included in our analysis. All eligible patients underwent a total of 12 h of double reverse osmosis water and standard bicarbonate dialysis per week (thrice-weekly) and were prospectively followed up to observe outcomes. The study protocol was approved by the institutional medical ethics committee of the Dalian Central Hospital, and all study patients provided written informed consent.

### Study variables

The age, sex, and cause of ESRD of each patient were obtained by electronic records form the hospital information system of Dalian Central Hospital. The causes of ESRD were divided into four categories in our study, including diabetes, glomerulonephritis, hypertension, and other diseases. Time on dialysis was defined as the duration of time between the first day of hemodialysis treatment and the first day of entry into the study. In December 2014, the blood samples of all included patients were taken before the mid-week maintenance HD session using standardized techniques and stored at − 80 °C. A range of laboratory parameters, including serum concentrations of hemoglobin, albumin, potassium, sodium, calcium, phosphorus, and alkaline phosphatase, were measured using standard procedures. Kt/V [(clearance of urea × dialysis time)/patient’s total body water] was used to estimate dialysis dose. In addition, standardized blood pressure measurements were performed before the mid-week maintenance HD session. Serum galectin-3 levels were detected by Human Galectin-3 Quantikine ELISA Kit (R&D Systems Inc., Minneapolis, MN, USA) in three circular wells following protocols provided for immunoassays, and the mean serum galectin-3 value was calculated as the final level. The assay range was 5.3–26.6 ng/ml, the mean intra-assay coeffificient of variation (CV) was 4.4%, the mean interassay CV was 6.0%.

### Outcomes and follow-up

We defined time to all-cause death as the primary endpoint and time to CV death as the secondary endpoint, respectively. All-cause mortality was death from any cause. CV mortality was defined as death directly associated with cardiovascular events (myocardial infarction, stroke) or sudden death or death occurring without evident cause (such as cancer, infection, traumatism). Patients were censored for lost to follow-up (December 31, 2019), end of the study period, or renal transplantation.

### Statistical analysis

Shapiro-Wilk W test was used to evaluate the normality of data. Continuous variables were expressed as mean ± standard deviation or median [interquartile range (IQR)], and intergroup comparisons were analyzed using t-tests for normally distributed data or the Mann-Whitney U tests for non-normally distributed data. Categorical variables were expressed as numbers and percentages, and differences between the two groups were examined using chi-square tests. Spearman’s rank correlation coefficient was used to assess the relationships between serum galectin-3 concentrations and other continuous variables.

Optimal cutoff points of galectin-3 were produced using X-tile software version 3.6.1 (Yale University School of Medicine, New Haven, CT, USA), [18] which identified the cutoff with the minimum *P* values from log-rank χ^2^ statistics for the categorical galectin-3 concentration in terms of survival [19]. Survival curves were generated using the Kaplan-Meier method, and differences between the curves for CV mortality were analyzed by using the log-rank test. Due to the violation of the proportional hazards assumption, the survival differences between two groups for all-cause mortality were ascertained using restricted mean survival time (RMST).

Unadjusted and multivariable adjusted hazard ratios (HRs) of mortality risks were calculated for two subsets of galectin-3 concentration (high vs low) based on the optimal cutoff point in the Cox regression model. We included the following covariates of interest based on previous studies and the availability of information: age, sex, time on dialysis, cause of ESRD, systolic/diastolic blood pressure, hemoglobin, albumin, Kt/V, alkaline phosphatase, potassium, sodium, calcium, phosphorus. Covariates for adjustment were selected based on a 10% change-in-estimate principle. The model included each covariate one at a time, and covariates were included as potential confounders in the final model if they changed the estimates by more than 10% or were significantly related to mortality risks. Schoenfeld residuals were used to test proportional hazards assumption. We additionally used the E-value methodology of VanderWeele and Ding to explore the potential for unmeasured confounding between galectin-3 and mortality in maintenance HD patients [20]. The E-value quantifies the required magnitude of an unmeasured cofounder that could negate the observe association [21]. Two-sided values of *P* <  0.05 were considered statistically significant. The data analysis was performed using STATA software version 11.2 (STATA Corp, College Station, TX).

## Results

As shown in Table 1, our study sample consisted of 506 eligible maintenance HD patients, including 270 males and 236 females. The median (IQR) age of the whole patient population was 58 (47–66) years. The median (IQR) duration of maintenance HD treatment was 51 (25–77) months, and glomerulonephritis was the most frequent cause of ESRD (40.5%). One hundred and eighty-eight maintenance HD patients (37.2%) died during 60 months of follow-up, 125 of which (66.5%) were CV deaths. Based on the X-tile software, we determined that the optimal cutoff point of galectin-3 for all-cause and CV deaths occurred at 8.65 ng/ml and divided the patient population into 2 subsets (galectin-3 >  8.65 ng/ml and galectin-3 ≤ 8.65 ng/ml). Baseline characteristics of the study population according to the optimal cutoff point are presented in Table 1. Except for pre-HD systolic blood pressure, the proportion of the other selected characteristics did not differ between the two groups. Table 2 presents the Spearman’s rank correlation coefficients between galectin-3 and a series of bio-clinical parameters in maintenance HD patients at baseline. We only observed a weak correlation between galectin-3 and systolic blood pressure (ρ = 0.088, *p* <  0.05).

**Table 1:** Baseline characteristics of the study patients according to the optimal cutoff point of galectin–3

Characteristic	Study population (***n*** = 506)	Galectin–3 (ng/ml)		
		≤ 8.65 (***n*** = 79)	> 8.65 (***n*** = 427)	***P***
Age, years	58 (47–66)	56 (43–63)	58 (48–66)	0.07
Male, n (%)	270 (53.4)	43 (54.4)	227 (53.2)	0.84
Time on dialysis, months	51 (25–77)	54 (31–71)	48 (24–79)	0.44
Cause of end–stage renal disease, n (%)				0.16
Diabetes	133 (26.3)	22 (27.8)	111 (26.0)	
Glomerulonephritis	205 (40.5)	28 (35.4)	177 (41.5)	
Hypertension	94 (18. 6)	21 (26.6)	73 (17.1)	
Other	74 (14.6)	8 (10.1)	66 (15.5)	
Systolic blood pressure, mmHg	150 (135–160)	140 (125–155)	150 (140–160)	< 0.05
Diastolic blood pressure, mmHg	80 (80–90)	80 (80–90)	80 (80–90)	0.07
Hemoglobin, g/l	110 (101–118)	111 (100–118)	110 (101–118)	0.49
Albumin, g/l	40.7 (39.0–42.4)	40.7 (38.9–41.9)	40.7 (39.0–42.5)	0.66
Single–pool Kt/V	1.37 (1.22–1.53)	1.38 (1.22–1.55)	1.37 (1.22–1.53)	0.92
Alkaline phosphatase, u/l	85 (69–117)	86 (72–118)	85 (69–117)	0.78
Potassium, mmol/l	4.96 (4.42–5.56)	5.01 (4.51–5.40)	4.96 (4.41–5.58)	0.85
Sodium, mmol/l	134.0 (131.8–136.3)	134.5 (131.9–135.8)	133.9 (131.8–136.4)	0.88
Calcium, mmol/l	2.36 ± 0.17	2.38 ± 0.17	2.36 ± 0.17	0.34
Phosphorus, mmol/l	1.99 (1.58–2.39)	1.94 (1.55–2.36)	1.99 (1.61–2.40)	0.28

*Note*: Data are displayed as mean ± standard deviation or median [quartile1, quartile3] for continuous variables and number (percent) for categorical variables

**Table 2:** Correlations of galectin–3 with other continuous variables

Characteristic	Galectin–3	
	ρ	***P***–value
Age	0.008	0.86
Time on dialysis	0.036	0.42
Systolic blood pressure	0.088	< 0.05
Diastolic blood pressure	0.048	0.28
Hemoglobin	−0.051	0.25
Albumin	0.038	0.39
Single–pool Kt/V	0.022	0.62
Alkaline phosphatase	< 0.001	0.99
Potassium	−0.022	0.62
Sodium	0.056	0.21
Calcium	−0.013	0.77
Phosphorus	0.034	0.45

**Fig. 1:**
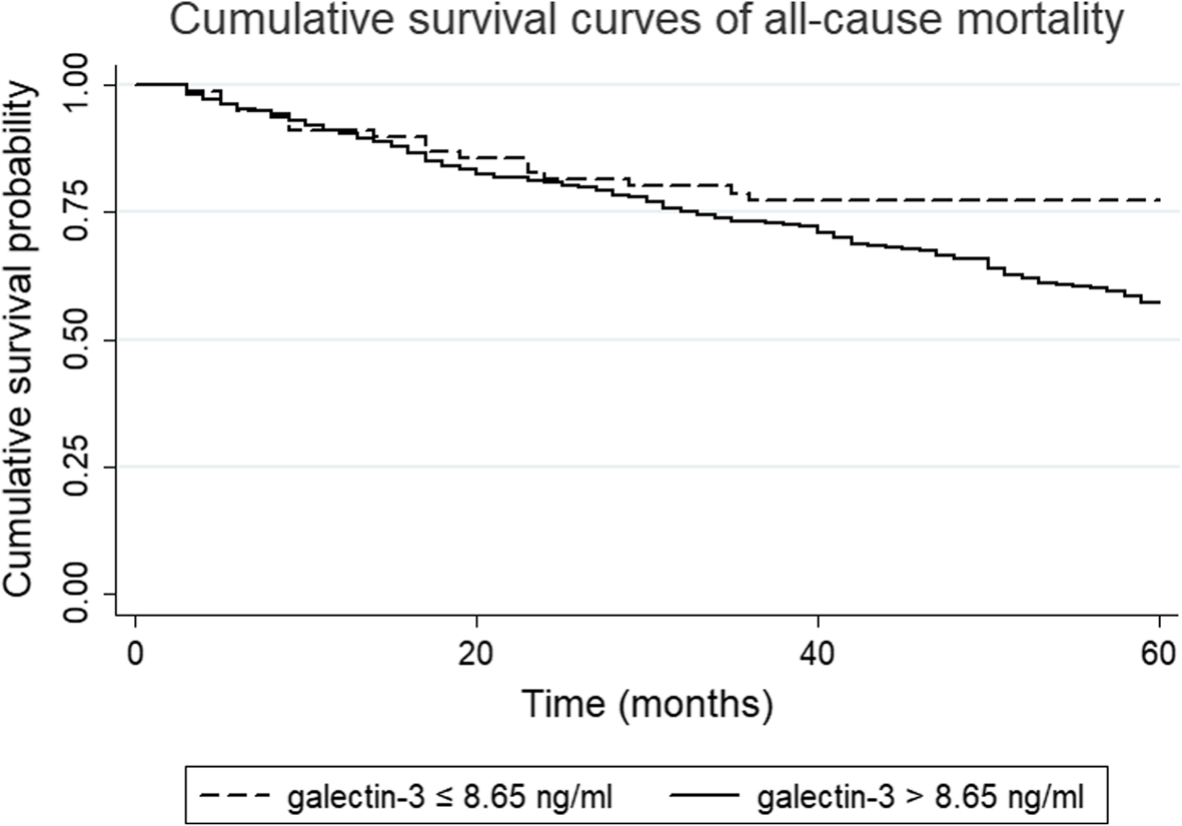
Kaplan-Meier survival estimates of all-cause mortality between the two galectin-3 subgroups divided by the optimal cutoff value generated by the X-tile program

**Fig. 2:**
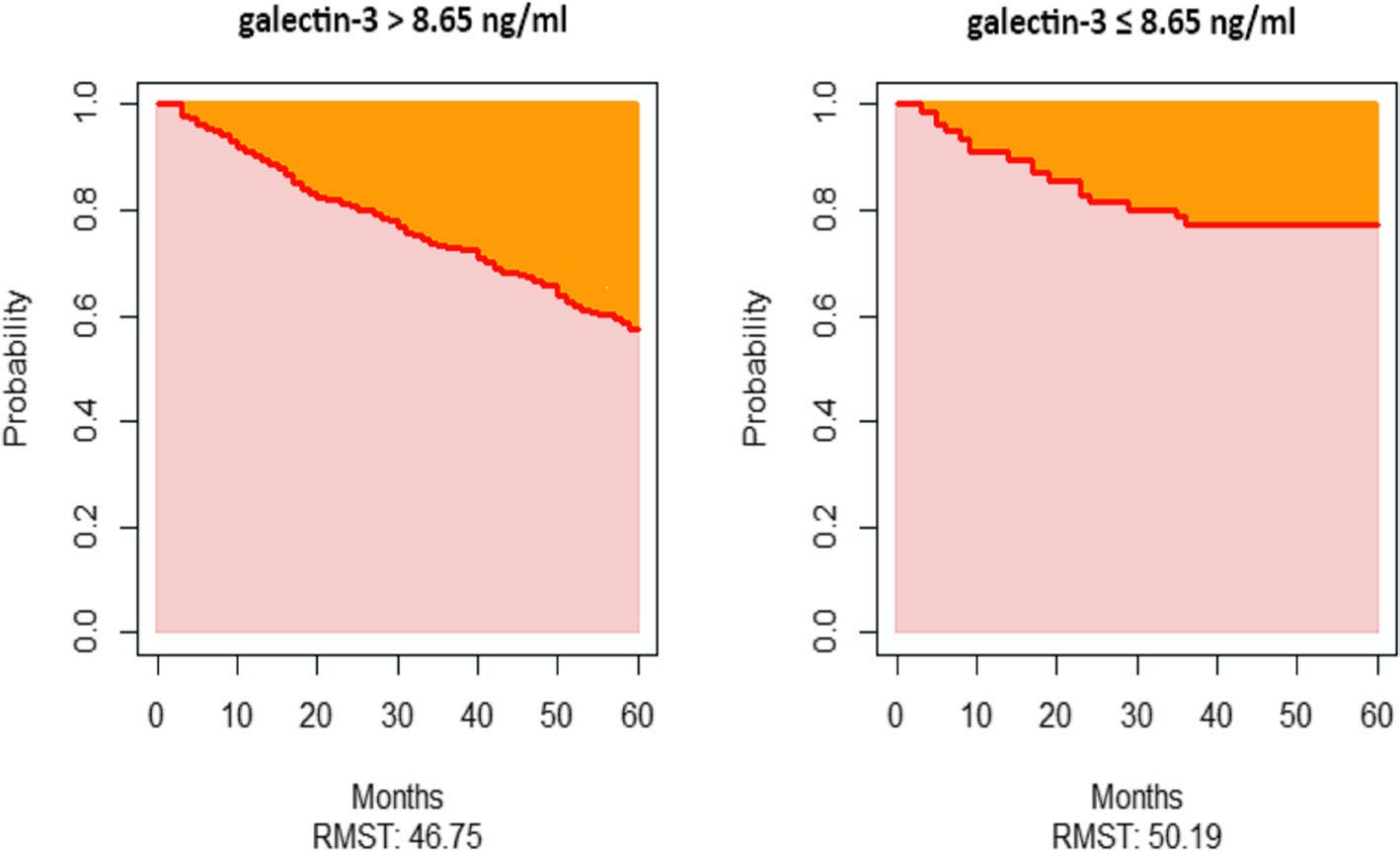
Restricted mean survival time for overall survival over 60 months. Restricted mean survival time (RMST) is the average time-to-event over a fixed follow-up period (60 months) and corresponds to the area under the survival curve. Over 60 months, maintenance hemodialysis patients with galectin-3 > 8.65 ng/ml lived, on average, 46.75 months; this was 3.44 months shorter than those with galectin-3 ≤ 8.65 ng/ml, who lived, on average, 50.19 months

**Fig. 3:**
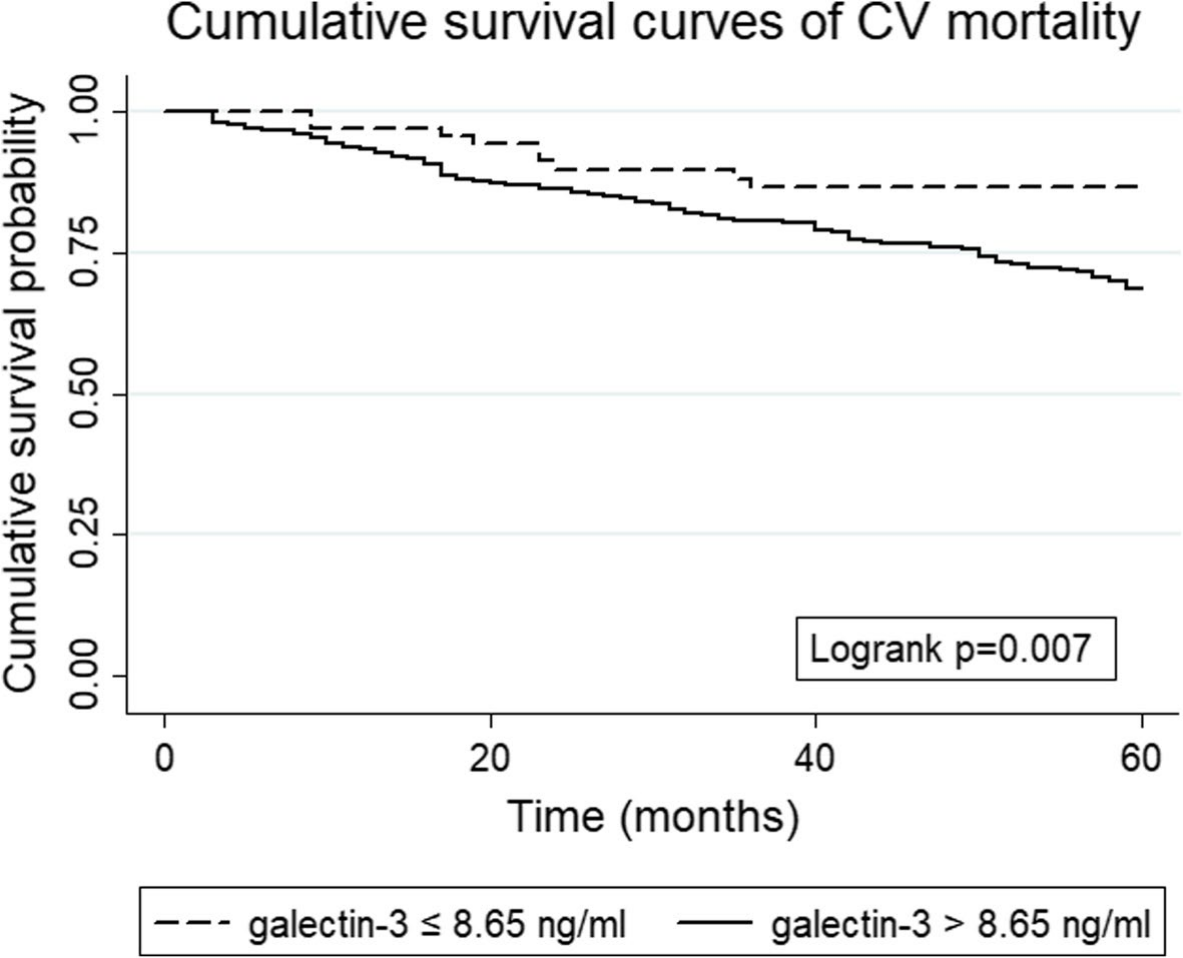
Kaplan-Meier survival estimates of cardiovascular mortality between the two galectin-3 subgroups divided by the optimal cutoff value generated by the X-tile program

Kaplan-Meier analysis was performed to assess the association between galectin-3 and all-cause mortality (Fig. 1). However, due to the crossing-curves problem, an alternative is to use RMST difference to quantify the treatment effect, and RMST refers to the area under the curve (Fig. 2). Over 60 months of follow-up, maintenance HD patients with galectin-3 >  8.65 ng/ml survived for 46.75 months on average; this was 3.44 months [95% confidence interval (CI), − 8.05 to 1.16, *p* = 0.14] shorter than those with galectin-3 ≤ 8.65 ng/ml, who survival for 50.19 months on average (Fig. 2). As shown in Table 3, higher baseline serum galectin-3 concentrations were associated with an increased risk of death in unadjusted analyses. In the fully adjusted model including age, ESRD due to diabetes, glomerulonephritis, and hypertension, hemoglobin, albumin, Kt/V, alkaline phosphatase, and sodium, maintenance HD patients with high galectin-3 concentrations had an increased risk of death (HR = 1.59, 95% CI 0.96–2.65, *p* = 0.07), but this was not statistically significant (Table 3). Similarly, no significant association was observed when serum galectin-3 as a continuous variable in these two models. The Kaplan-Meier curve of CV mortality showed that maintenance HD patients with galectin-3 > 8.65 ng/ml had a lower probability of survival compared with those with galectin-3 ≤ 8.65 ng/ml (log-rank *p* <  0.05, Fig. 3). The univariate analysis of galectin-3 effect on CV mortality in maintenance HD population found that high galectin-3 groups had an increased risk of CV mortality (HR = 2.47, 95% CI 1.25–4.87, Table 4). We also found that the following covariates were associated with CV mortality: age, ESRD due to diabetes, glomerulonephritis, and hypertension, albumin, Kt/V, alkaline phosphatase, and sodium in univariate analysis with Cox regression (Table 4). After adjustment, higher serum galectin-3 concentrations were significantly associated with a 113% increase in CV mortality (HR = 2.13, 95% CI 1.07–4.26, *p* <  0.05, Table 4). However, we did not observe statistically significant association when serum galectin-3 as a continuous variable in these two models. The E-values for the point estimate and lower confidence bound for CV mortality were 2.76 and 1.27, respectively.

**Table 3:** Univariate and multivariable Cox regression analysis of prognostic factors for all–cause mortality

Characteristic	Cox Univariate		Cox Multivariate			
	HR (95% CI)	***P***–value	HR (95% CI)	***P***–value	HR (95% CI)	***P***–value
Galectin–3 (> 8.65 versus ≤8.65 ng/ml)	1.92 (1.17–3.17)	0.01	1.59 (0.96–2.65)	0.07	–	–
Galectin–3 (continuous variable)	0.99 (0.97–1.02)	0.70	–	–	0.99 (0.96–1.03)	0.74
Age (years)	1.07 (1.05–1.08)	*P* < 0.01	1.06 (1.04–1.07)	*P* < 0.01	1.06 (1.04–1.08)	*P* < 0.01
ESRD due to diabetes (yes versus no)	2.67 (2.00–3.57)	*P* < 0.01	2.20 (1.34–3.59)	*P* < 0.01	2.12 (1.30–3.46)	*P* < 0.01
ESRD due to glomerulonephritis (yes versus no)	0.33 (0.23–0.47)	*P* < 0.01				
ESRD due to hypertension (yes versus no)	1.50 (1.08–2.10)	0.02				
Hemoglobin (g/l)	0.99 (0.98–1.00)	0.02				
Albumin (g/l)	0.85 (0.81–0.89)	*P* < 0.01	0.93 (0.88–0.99)	0.01	0.93 (0.88–0.98)	0.01
Dialysis dose (spKt/V)	0.45 (0.24–0.85)	0.01	0.38 (0.19–0.77)	*P* < 0.01	0.37 (0.18–0.75)	*P* < 0.01
Alkaline phosphatase (u/l)	1.003 (1.001–1.004)	*P* < 0.01	1.004 (1.002–1.005)	*P* < 0.01	1.004 (1.002–1.005)	*P* < 0.01
Sodium (mmol/l)	0.93 (0.89–0.97)	*P* < 0.01				

*Abbreviations*: *CI* confidence interval, *ESRD* end–stage renal disease, *HR* hazard ratio

**Table 4:** Univariate and multivariable Cox regression analysis of prognostic factors for cardiovascular mortality

Characteristic	Cox Univariate		Cox Multivariate			
	HR (95% CI)	***P***–value	HR (95% CI)	***P***–value	HR (95% CI)	***P***–value
Galectin–3 (> 8.65 versus ≤8.65 ng/ml)	2.47 (1.25–4.87)	P < 0.01	2.13 (1.07–4.26)	0.03	–	–
Galectin–3 (continuous variable)	1.01 (0.97–1.05)	0.61	–	–	1.01 (0.98–1.05)	0.49
Age (years)	1.06 (1.05–1.08)	P < 0.01	1.05 (1.03–1.07)	P < 0.01	1.05 (1.04–1.07)	P < 0.01
ESRD due to diabetes (yes versus no)	2.66 (1.86–3.80)	P < 0.01	2.52 (1.35–4.72)	P < 0.01	2.43 (1.30–4.53)	P < 0.01
ESRD due to glomerulonephritis (yes versus no)	0.35 (0.23–0.54)	P < 0.01				
ESRD due to hypertension (yes versus no)	1.53 (1.02–2.30)	0.04	2.01 (1.04–3.88)	0.04		
Albumin (g/l)	0.85 (0.80–0.90)	P < 0.01	0.91 (0.85–0.97)	P < 0.01	0.91 (0.85–0.97)	P < 0.01
Single–pool Kt/V	0.38 (0.18–0.83)	0.01	0.30 (0.12–0.72)	P < 0.01	0.29 (0.12–0.69)	P < 0.01
Alkaline phosphatase (u/l)	1.003 (1.001–1.004)	0.001	1.004 (1.002–1.005)	P < 0.01	1.004 (1.002–1.005)	P < 0.01
Sodium (mmol/l)	0.92 (0.88–0.97)	P < 0.01				

*Abbreviations*: *CI* confidence interval, *ESRD* end–stage renal disease, *HR* hazard ratio

## Discussion

In this prospective Chinese cohort of 506 maintenance HD patients followed for up to 60 months, we found that elevated galectin-3 concentrations were associated with increased risks for CV mortality instead of all-cause mortality. Multivariable analysis results persisted even after following a 10% change-in-estimate principle. Galectin-3 may be a promising marker for CV risk stratification in maintenance HD patients.

Galectin-3 is expressed predominantly by activated macrophages and involved in multiple pathological processes, including fibrosis, inflammation, and tumor growth [13]. Accumulating evidence indicates that galectin-3 plays a key role in fibrogenesis in different organ systems, including liver, kidney, lung, and myocardial [22]. Increased serum galectin-3 levels activate a variety of profibrotic factors and induce cardiac fibroblasts to proliferate and transform, contributing to myocardial fibrosis and adverse remodeling [13]. In addition to acting as a direct profibrotic agent, galectin-3 also mediates aldosterone-induced cardiac, vascular, and renal fibrosis [23]. Since progressive cardiac fibrosis is an essential aspect in the evolution of cardiac dysfunction and a substrate for lethal arrhythmias and sudden death, [24] it is intuitive that a blood marker of cardiac fibrosis would be a prognostic factor for survival and CV events [25].

A recent meta-analysis [26] of four epidemiologic studies [1, 15–17] found no association between higher serum levels of galectin-3 and the risk of all-cause death in maintenance HD patients (HR = 1.17, 95% CI 0.96–1.42). This is consistent with our findings that galectin-3 is not a sensitive biomarker for all-cause mortality in maintenance HD patients. In addition, the meta-analysis of two studies [15, 17] also supports our results based on the secondary endpoint and suggests that galectin-3 may be a reliable predictor of CV mortality in maintenance HD patients (HR = 1.06, 95% CI 1.00–1.13). Among patients with ESRD, 40 to 50% of deaths have been attributed to CV disease [27–29]. Several studies have shown that CV mortality is the leading cause of death in patients receiving hemodialysis or peritoneal dialysis and is 10 to 20 times higher in this population than in the general population [30, 31]. Some biomarkers, such as serum triglyceride to high-density lipoprotein cholesterol ratio, [32] N-terminal pro-B-type natriuretic peptide, [33] and uric acid, [34] have been found to have predictive values for CV mortality in maintenance HD patients. In a study involving 423 HD patients, [1] there were 78 composite outcomes (a composite of all-cause death or cerebrocardiovascular events) during a mean follow-up of 2.1 ± 0.4 years. The results showed that galectin-3 was significantly associated with the composite outcome [1]. To date, only two prior studies [15, 17] with inconsistent results have examined the association of galectin-3 with CV mortality in maintenance HD patients. In a prospective cohort study of 86 adults on HD in Taiwan province, China, Ko et al. did not find an association between CV mortality and galectin-3 levels (HR = 1.04, 95% CI 0.99–1.10) after adjusting for age, C-reactive protein, albumin, normalized protein catabolic rate, vascular cell adhesion molecule 1, and smoking [15]. Although the HD population (taking into account age, gender composition, and ethnicity) was homogeneous with our study, sample size, adjustment factors, and determination method of the optimal cutoff point of galectin-3 could account for inconsistent findings to some extent. Notably, the galectin-3 level cutoff points were produced using the X-tile program in our study, which identified the cutoff with the minimum *P* values from log-rank χ^2^ statistics for the categorical galectin-3 level in terms of survival [19]. However, in Ko et al.’s study, [15] the cutoff point was simply determined based on the mean galectin-3 level of the whole study population. In a post hoc analysis of the 4D Study (Die Deutsche Diabetes Dialyse Studie), Drechsler et.al focused on 1168 dialysis patients with type 2 diabetes mellitus followed for 4 years and found that log-transformed galectin-3 level was associated with CV events defined as a composite of cardiac death in both unadjusted (HR = 1.13, 95% CI 1.03–1.24) and fully adjusted (HR = 1.12, 95% CI 1.01–1.24) models [17]. Patients suffering from type 2 diabetes mellitus undergoing dialysis were enrolled in this cohort; as a result, the findings may not be generalizable to the overall HD population. In the present study, participants had multiple causes of ESRD, making our findings generalizable to the overall maintenance HD population at large.

Our study has several strengths. A main advantage is the relatively large sample size, which allows us to explore the associations between galectin-3 level and all-cause and CV mortality in maintenance HD patients in a more statistically precise manner. Second, in our study, predefined outcomes including both all-cause and CV mortality were prospectively observed over a relatively long-term follow-up (60 months). Third, as mentioned earlier, we used the method described by Camp et al. to determine the optimal cutoff point for galectin-3. In addition, the RMST difference was used to detect a true treatment effect when there was a crossing-curves problem [35, 36]. Finally, according to a 10% change-in-estimate principle, our analyses were adjusted for a large number of potential confounders.

There are several study limitations to be considered in the interpretation of our study findings. First, in our study, only baseline galectin-3 levels were obtained and we did not have serial measurement of galectin-3 over time. Single-point measurement may not reflect substantial intra-individual variability over time and may increase the probability of random measurement error. On the other hand, incomplete adjudication of cause of death may have led to misattribution of CV deaths as noncardiovascular. Second, our HD patients were all from the same Hospital in China and whether the findings of the present study can be extrapolated to patients of other countries is unclear. In addition, all maintenance HD patients were Asian in the present study, thus the generalizability of the study findings across ethnicities remains unclear. Third, due to the unavailability of data, several traditional and non-traditional risk factors for CV death, such as serum high sensitivity C-reactive protein, smoking, nutritional parameters and residual renal function or urine volume [37], were not adjusted in multivariable models. Therefore, as with other studies, our study may be limited by residual confounding. However, the E-value sensitivity analysis suggested that the observed HR of 2.13 for CV mortality could only be explained by an unmeasured confounder that was associated with both galectin-3 and risk of CV death by a risk ratio of more than 2.76 above and beyond that of the confounders that were measured in the present study. Therefore, it is implausible that an unmeasured confounder exists than can negate the effect of galectin-3 observed in the current analysis study. Fourth, because we did not have data on other cardiac biomarkers, such as N-terminal pro-B-type natriuretic peptide and troponin etc., combined analyses were not possible in our study. Finally, considering the observational nature of study design, our findings cannot show causality between galectin-3 and CV death in patients on maintenance HD. However, we add new epidemiological evidence that galectin-3 may be a novel biomarker for CV risk stratification in patients receiving HD treatment.

## Conclusion

In summary, higher blood levels of galectin-3 were significantly associated with an increased risk of CV mortality in patients with ESRD who are on maintenance HD. Given the importance of early detection of high-risk maintenance HD patients, we provide evidence that galectin-3 may be an independent predictor of CV mortality in maintenance HD patients.

## Data Availability

The datasets used and/or analyzed during the current study are available from the corresponding author upon reasonable request.

## Abbreviations

HD: hemodialysis
CV: cardiovascular
HRs: Hazard ratios
CIs: confidence intervals
ESRD: End-stage renal disease
HF: heart failure
IQR: interquartile range
RMST: restricted mean survival time.
